# Postpartum depression-an exploratory mixed method study for developing an indigenous tool

**DOI:** 10.1186/s12884-023-06192-2

**Published:** 2024-01-10

**Authors:** Maham Abdullah, Shirmeen Ijaz, Shamaila Asad

**Affiliations:** Riphah International University, Lahore, Pakistan

**Keywords:** Postpartum depression, Indigenous tool, Motherhood, Validation studies, Low- and middle-income countries, Pakistan, Mixed methodology

## Abstract

**Background and objective:**

Motherhood is considered to be a joyous occasion cross-culturally but can bring forth an array of issues including depression. In Asia, Pakistan is dangerously high on the prevalence of postpartum depression (PPD) with sporadic results ranging from 28 to 63%, which could be due to the use of non-indigenous tools.

**Methods:**

An exploratory study-mixed method design was implied. During phase I mothers in the postpartum phase (up to 12 months) and experts were interviewed, items were generated, and pilot study was conducted. In second phase exploratory and confirmatory factor analysis was conducted to establish construct validity, convergent and divergent validity was also established.

**Results:**

A pool of 46 items was generated related to postpartum depression, which was reduced to 35 items as per the factor loading of 0.5 in exploratory factor analysis. Psychometric properties of the scale revealed good reliability (Cronbach α = 0.92) and factor structure of the scale. As per exploratory factor analysis five factors were revealed, explaining 58.07% variance, and the model was confirmed by confirmatory factor analysis. The scale showed significant positive correlation with Edinburgh' postpartum depression scale, depression anxiety and stress scale; establishing convergent validity and significant negative correlation with satisfaction with life scale; establishing divergent validity of the scale.

**Conclusion and implications for translation:**

Questionnaire of postpartum depression scale is a reliable and valid tool that can be used to measure postpartum depression in Asian mothers and provide targeted interventions.

## Introduction

Motherhood is perceived to be an occasion of elated emotions. However, it brings forth an experience of not only joy but can extend to sadness, feeling lost and depressed. Evidence suggests that postpartum depression (PPD) is linked with profound and incessant ramifications affecting interactions between mother and infant, resulting in issues with maternal bonding [1], breastfeeding and role of mothers [2]. The repercussions can also be seen on physical and psychological health of mothers, occupational functioning and risky behaviors [3]. For infants the extensive consequences of PPD are not limited to only early developmental stages but continue to influence development in adolescence and adulthood including atypical neurodevelopment [4] anthropometry, language, physical health, motor, emotional and social functioning [5].

According to American Psychiatric Association [6], the diagnostic criteria of postpartum (right after birth till one year) depression include; “sad mood, loss of interest or pleasure, changes in sleep and appetite, frequent crying without reason, increased fatigue, inability to concentrate or make decisions, increase in purposeless physical activity, feeling of hopelessness and guilt, suicidal ideation, trouble bonding with the baby, feelings of being a bad mother and fear of harming the baby”.

Diagnostic criteria like these stems from the construct of postpartum depression which has been conceptualized in the West and has been measured by using assessment tools constructed predominantly in the West, whereas Asian cultures vary in great deal to the west. These western tools have been used in other cultures as well, the rationale being the universality of psychological disorders, like postpartum depression. However, the errors behind such assessment cannot be dismissed due to the differences in local norms and values, creating methodological issues and rendering questions on the validity of the results [7].

Although such issues can be dealt through translation and adaptation of these tools according to culture, it still does not cater to all the cultural factors, as the understanding of the etiology, symptoms of disorders and their symptomatic expression varies within cultures. The emotions are pan-cultural, but the experience and expression of emotions depends upon various reasons like the words available in a particular language or the socio-economic factors etc. For instance, the sample taken in Western studies are predominantly living in nuclear families, educated, financially independent, whereas, in Asian countries like India and Pakistan, people live in Joint families, and this is one of the main themes linked with postpartum depression, e.g., joint-family system, relationship with mother in- law, unsupportive in-laws, uncaring husband, overwhelmed with domestic responsibilities etc. [8].

The above-mentioned cultural factors are not accounted for in the western tools that are translated in indigenous studies for measuring postpartum depression. Therefore, in order to understand a disorder and to assess it, conceptual organization of cultural knowledge should be credited. Which means to understand how the general population talks about the illness in their social and personal context, as cultural differences create differences in the manifestation of the illness. Studies have reported that non-western populations tend to focus less on psychological and mental symptoms of a disorder as compared to western populations. Similarly, non-western populations as well as the Pakistani population tend to focus more on somatic symptoms and explain their illness through somatization.

Furthermore, agreeing to a few gauges, PPD could be a major issue in Pakistan with predominance extending from 28.8% [9] to 36% [10] to 94% [11] in spite of evaluating postpartum depression over the same time frame of three months postpartum. Rahman and Creed [11] conducted a longitudinal study in Rawalpindi, Pakistan according to which 62% of depressed women were found to be depressed at 12 months of postpartum. Asian countries account for variations from 3.5% to 63.3% [7] and in Pakistan it is 63% [12]. One reason for these sporadic prevalence rates could be the use of depression tools in the studies that are not specialized to measure depression in the postpartum phase like Beck Depression Inventory. Another reason could be the use of non-indigenous cultural tools like Edinburgh's Postpartum Depression Scale (EPDS) that does not account for the screening of unique cultural occurrences of Pakistani culture. Also, limitations of using Edinburgh's postpartum depression scale are that it does not evaluate the experiences of the new mother or factors such as feeling of losing control, isolation, feelings of un realness and irritation, losing oneself and difficulties with concentration [13], which makes it difficult to provide targeted interventions for postpartum mothers experiencing depression. Therefore, it is important to develop a scale that can measure postpartum depression, accounting for the range of experiences felt by mothers in the postpartum phase, which can be used in other cultures as well.

## Objectives

- To develop an indigenous scale to measure postpartum depression.
- To establish psychometric properties of the scale.

## Methods

Exploratory Research Design was used in this study, which is a sub type of Mixed Method designs. In phase I qualitative interviews were taken from experts and target population (*n*=12). Mothers in the postpartum phase up to 12 months were included in the study to cover all phases of postpartum, through purposive sampling. Data was collected from them in two different points (from 4 to 6 weeks and 9 to 12 weeks) to achieve accurate experiential representation of depression specific to postpartum phase [14] in Pakistani mothers. For development of scale, the mothers in the postpartum phase were interviewed and their responses were recorded. Afterwards, content analysis was done to generate themes. The themes that emerged from qualitative interviews were feelings of isolation and abandonment. Many individuals expressed struggles in meeting their commitments. A sense of disconnection from one's identity emerged as a common theme. Participants often recounted experiences of maternal inadequacy. Anger and frustration were frequently reported. Loss of enjoyment and engagement were evident in participants' narratives. Bodily complaints and physical ailments were mentioned. Disturbances in sleep patterns and appetite were observed. Uncontrollable episodes of crying were reported. Irritability and persistent fatigue were a pervasive challenge. Participants voiced concerns about social judgment and scrutiny.

After generating themes, items were developed. Items were also developed after reviewing other postpartum depression scales like Edinburgh's Postpartum Depression Scale and reviewing literature. These items were reviewed by 6 experts through the item content validity index (ICVI). Only the items with greater than .79 ratings were incorporated in the final scale for pilot testing, which were 46 statements and responses were taken on a Likert scale ranging from 1 (not at all) to 4 (Most of the times).

Pilot testing was conducted (*n*-50). Open-ended feedback was also taken to get valuable information. It allowed for refinement of the tool in regard to its features like salience, variance, phraseology, order of items and if there was any ambiguity in item wording. Preliminary analysis (Cronbach alpha) was done, which was .90 establishing good reliability of the items.

A Demographic sheet was prepared, and data was collected from 315 Pakistani mothers in postpartum period, with their consent and then analysis was done for reliability and exploratory factor analysis and confirmatory factor analysis was done on 392 participants. Participants (*n*=100) also took Edinburg’s Postpartum Depression Scale, Depression Anxiety and Stress Scale for establishing convergent validity and Satisfaction with life Scale for establishing divergent Validity.

### Statistical analysis

The data analysis included the assessment of the structural (underlying factors or subscales) and psychometric (reliability and validity) properties of the questionnaire. The conventional methods were performed using exploratory factor analysis and confirmatory factor analysis for construct validity and Pearson moment product correlation for internal consistency, convergent and divergent validity. EFA and Pearson moment product correlation were conducted using SPSS and CFA was done through AMOS.

## Results

Table 1 presents demographic characteristics of the sample (n-315). Mean age of the participants was 27.55 with a standard deviation of 4.73. Participants were 315 mothers in the postpartum phase. Most of the participants were married (86%) in this study. Most of the participants were graduates (68%). Many participants belonged to middle class families (86.3%) and the nuclear family system (51.7%). The mean of an individual's monthly income was 107485.71 and standard deviation 86512.81. In the present study 17.5% participants had vaginal delivery (without medical intervention), 27.3% participants had vaginal delivery (assistant delivery), 38.1% participants had C-section and 17.1% participants had Emergency C-Section.

**Table 1:** Descriptive statistics of demographic variables of participants (*N*-315)

Variables	M (SD)	*f (%)*
Age	27.55 (4.73)	
Marital Status		
Married		271 (86.0%)
Divorced		7 (2.2%)
Separated		22 (7.0%)
Widowed		15 (4.8%)
Total		315 (100%)
Education		
Matric		1 (0.3%)
Intermediate		45 (14.2%)
Graduation/ Masters		216 (68.6%)
MS/M.Phil		53 (16.9%)
Total		315 (100%)
Family System		
Nuclear		163 (51.7%)
Joint		152 (48.3%)
Total		315 (100%)
Socioeconomic Status		
Lower Class		11 (3.5%)
Middle Class		272 (86.3%)
Upper Class		32 (10.2%)
Total		315 (100%)
Paid Occupation		
Yes		175 (55.6%)
No		140 (44.4%)
Total		315 (100%)
Family Income	107,485.71 (86,512.81)	
Residential Area		
Rural		95 (30.2%)
Urban		220 (69.8%)
Total		315 (100%)
Any Physical Problem		
Yes		33 (10.5%)
No		257 (81.6%))
Prefer not to say		25 (7.9%)
Total		315 (100%)
Birth of Infant		
Premature (less than 37 weeks)		58 (18.4%)
Term (in 37–42 weeks)		194 (61.6%)
Post term (more than 42 weeks)		63 (20.0%)
Total		315 (100%)
Mode of delivery		
Vaginal (without medical intervention)		55 (17.5%)
Vaginal birth (assistant delivery)		85 (27.3%)
C-Section		120 (38.1%)
Emergency C-Section		54 (17.1%)
Total		315 (100%)

*N* Number of participants, *M* Mean, *SD* Standard deviation, *%* Percentage, *f* Frequency

The present study aimed at development and validation of Questionnaire of Postpartum Depression (QPD) for postpartum population. Exploratory Factor Analysis (EFA) and Confirmatory Factor Analysis (CFA) was carried out to develop the factor structure of the QPD. Moreover, psychometric properties of the QPD were also established including reliability, convergent and divergent validity.

The Kaiser-Meyer-Olkin (KMO) was excellent at 0.95 [15] and Bartlett's test of sphericity was significant at *p* < 0.001(KMO index=0.96, χ2=8038.15, *P*=0.000) according to which exploratory factor analysis could be applied to the data set to obtain factors. Principal Component Analysis (PCA) with varimax rotation was run on a sample of 315 mothers. It was run a few times to determine the most appropriate factor numbers. Table 2 shows five factors were extracted based on The Scree-Plot elbow dip and Eigenvalues greater than one [14]. These factors explained a total variance of 58.07%. A total of 35 items were retained whose factor loading was greater than 0.5. Factor 1 (feeling lost and alone) had a total of 16 items which included statements like “I feel I am unable to understand anything” and “I think I am alone”. Factor 2 (suicidal ideation) had a total of 5 items like “I think of committing suicide”. Factor 3 (anger) consists of 5 items like “I feel very angry”. Factor 4 (somatic symptoms) includes 4 items like “Without any medical reason there is pain in my legs”. Factor 5 (anhedonia) consists of 5 items like “Nothing gives me pleasure”.

**Table 2:** *Exploratory factor analysis for pakistan postpartum depression scale by using principal component with varimax rotation (N* = *315)*

Items	Feeling lost and alone Items 16	Suicidal ideation Items 5	Anger Items 5	Somatic symptoms Items 4	Anhedonia Items 5
21	.63				
22	.63				
23	.65				
24	.65				
25	.62				
26	.64				
27	.53				
28	.54				
29	.52				
30	.66				
31	.58				
32	.56				
35	.70				
36	.75				
37	.68				
38	.73				
3		.61			
8		.76			
9		.73			
10		.77			
11		.70			
1			.54		
2			.52		
4			.70		
6			.65		
7			.62		
43				.79	
44				.74	
45				.79	
46				.76	
12					.57
13					.73
15					.58
16					.63
19					.57
Eigen Value	13.74	2.48	1.74	1.41	1.20
% Variance	39.26	7.10	4.97	4.01	3.43
Cum. %	29.26	46.36	51.33	55.34	58.77

The five factors of the tool had moderate to good internal consistency (α=0.59 to 0.90) and had low to moderate correlations (*r* = .25 to .38) revealing that they were not derived from a singular underlying latent variable. The overall scale had excellent reliability (α = 0.92). Confirmatory Factor Analysis (*N*=392) showed that the model was fit and was consistent with the empirical data based on the goodness-of-fit indices, mentioned in Table 3. According to Kline [16] required indices to be reported are mentioned along with recommended values. Comparative Fit Index (CFI) divided by degrees of freedom (df) is a goodness-of-fit index used to assess how well a hypothesized model fits the observed data. A CFI/df value of less than 3 is a good fit. The observed value of CFI/df=2.235 (which is good). Root Mean Square Error of Approximation (RMSEA) compares hypothesized models to perfect models. Value of RMSEA less than 0.05 is better fit and less than 0.08 is acceptable. In this study the observed value of RMSEA=0.05 (which is moderate). Goodness-of-fit index (GFI) measures proportion of variance in the observed covariance matrix and its value ranges from 0 to 1, the higher the value the better the fit index. GFI value was 0.84 (the higher the better). Adjusted Index of Goodness of Fit (AGFI) is an adaptation of the Goodness of Fit Index (GFI) that considers how many indicators a confirmatory factor analysis (CFA) model has for each latent variable. Its value ranges between 0 to 1, the closer the value is to 1 the better fit between model and data. The value of AGFI of this study is 0 .81(the higher the better). In confirmatory factor analysis (CFA), Standardized Root Mean Square Residual (SRMR) is a goodness-of-fit index that evaluates how well a proposed model fits the observed data. Since there are no links between the variables in an independence model, the SRMR, which is a relative fit index, compares the proposed model to that model. A better match is indicated by a lower SRMR value; values less than 0.08 are regarded as appropriate. The observed value of SRMR was shown to be 0.06 (acceptable, as it should be <0.09).

**Table 3:** Confirmatory Factor analysis of model 1 of Questionnaire of postpartum depression

Model	CMIN/df	GFI	AGFI	SRMR	RMSEA
Model 1 of 35 items	2.235	0.84	0.81	0.06	0.05

Table 4 shows significant positive correlation of “questionnaire of postpartum depression” with Edinburgh's postpartum depression scale and depression anxiety and stress scale, and significant negative correlation with satisfaction with life scale establishing convergent and divergent reliability.

**Table 4:** Correlation Matrix for all the Variables Used in the Study (N-100)

Variables	1	2	3	4
QPD	–	.63**	.43**	-.58**
EPDS	–	–	.44**	-.54**
DAS	–	–	–	-.28**
SWL	–	–	–	–-

*QPD* Questionnaire of Postpartum Depression, *EPDS* Edinburg’s Postpartum Depression scale, *DAS* Depression Anxiety Stress Scale, *SWL* Satisfaction with Life Scale

**p < .001

## Discussion

A culturally sensitive measure called the Questionnaire for Postpartum Depression (QPD) was created to evaluate postpartum depression (PPD) in Pakistani women. It tackles a number of shortcomings of the PPD screening instruments now in use, specifically those of Beck and Gable's Postpartum Depression Screening Scale (PDSS) and Edinburgh Postnatal Depression Scale (EPDS). Because PDSS is a western-based diagnostic instrument, it is limited in its applicability to Pakistan's non-English speaking population and is unable to determine the local manifestation of depression.

EPDS has trouble identifying the subtle cultural differences associated with PPD in Asian countries like Pakistan. Two major issues with using the EPDS in this situation are overdiagnosis and insensitivity to cultural symptom presentations. Moreover, in Pakistan, the EPDS is the most often utilized instrument for PPD evaluation. Additionally, that is only for research and not for the normal clinical assessment of new moms, which is mandated by the World Health Organization [17]. The Edinburgh Postnatal Depression Scale research trend from the 1990s to the present, as per review of literature. Furthermore, these studies were never able to assess and record the mother's experiences as part of postpartum depression. Symptoms like loneliness, confusion, irritability and somatic complaints are not part of this assessment.

These issues are addressed by the creation of an indigenous instrument such as the QPD (Questionnaire for Postpartum Depression), which includes culturally appropriate items that represent the unique experiences and symptom presentation of Pakistani women. One significant example of this cultural sensitivity is the inclusion of somatic symptoms, which have a higher cultural value in Pakistani society, as here more validation is given if symptoms are physical in nature. These results are consistent with the findings of Ahmed and Khan [18]. Physical health is highly regarded in Pakistani society and is perceived as a reflection of one's general health and well-being. The focus on treating physical illnesses arises from the fact that somatic symptoms are frequently linked to bodily rather than mental discomfort [18]. This may conceal underlying emotional problems and postpone the diagnosis of PPD.

Asian countries, like Pakistan are collectivistic, due to which it was believed that the support systems exempt Asian mothers to develop PPD but the research indicates otherwise and one of the factors of QPD is of “feeling lost and alone” which could be due to the fact that psychological issues are hushed and stigmatized, which is one the main reasons of not getting the help which is available even if limited.

Another emerged factor was “anger”, although anger isn't usually seen as a primary symptom of PPD from a Western perspective, there are a number of cultural elements that can make rage a key symptom for Pakistani women. These results are consistent with the study done by Ali et al in 2015. In Pakistani culture, expressing anger directly might be frowned upon or viewed as socially inappropriate, especially for women [19]. Internalized rage and frustration brought on by this emotion suppression can show up as irritability, mood swings, or outbursts. The focus placed in the culture on upholding social harmony and putting up a happy front may impede candid discussions regarding emotional difficulties, such as irritation and wrath. It might be more difficult for women to express their emotions in a healthy way and increase the intensity of these feelings when they lack emotional support and an outlet. This makes it crucial to screen women in the postpartum phase so that they can be treated effectively without progression to full blown disorder.

In this study, a new tool called the QPD has been created and tested by researchers to measure postpartum depression. The results showed that the QPD is a reliable and valid tool for measuring postpartum depression. These findings suggest that the QPD could be useful for identifying and addressing postpartum depression in mothers. As this is developed in the national language, which is Urdu, it will be easier to understand for the nationals and hence provides a true picture of postpartum depression.

As hypothesized, the results confirmed that the QPD tool showed a significant positive correlation with other measures of depression that were theoretically related, indicating its convergent validity. Furthermore, the tool showed a significant negative relationship with non-measures of depression, indicating initial evidence of divergent validity.

It is a continuous work to validate a tool and there are still several areas that need to be explored to further develop and evaluate the QPD tool. This study lacked sampling control because a sophisticated sampling design was not employed. Additionally, the majority of the participants were married, non-working women from different regions of Pakistan without equal representation. Therefore, it is essential to establish the psychometric properties of the QPD tool in diverse samples, especially those who are susceptible to developing postpartum depression. Doing so will ensure that the tool is effective and reliable in identifying postpartum depression in different populations. Albeit, the mothers in postpartum phase accepted the measure in its current form which could be due to the qualitative investigation used for its development with alpha correlation of 0.92.

### Strengths and limitations of the study

Diverse samples from different cities of Pakistan was a strength. Also, validation of the scale yielded excellent reliability and validity. Like any other study in social science, the current study also elucidated certain limitations that should be conceived as suggestions as well. Study sample was less, more sample size is advised for better factorial structure. Study participants who participated were not true representatives as per the population, it is therefore suggested that future researchers may extend this sample to national level with equal proportions of representation in order to comprehend the postpartum symptoms with more precision. Because of limited resources and time constraints convenient purposive sampling technique was used, whereas use of more sophisticated sampling procedure like probability or cluster sampling procedures may yield more representative and refined factorial structure. More convergent and discriminant validity evidence to be ensured with diverse demographic samples as well as retest reliability should be established.

## Conclusion and implications

QPD is a psychometrically sound tool to measure depression in postpartum women in Pakistan and other Asian countries. It will be particularly useful for professionals working with the postpartum population to screen postpartum mothers for depression and provide targeted interventions based on the experiences and feelings reflected by the items. Also, by using this tool prevalence rates of postpartum depression can also be checked which might be able to cater to the sporadic prevalence rates and provide a true picture that can help in making policies at national level.

## Data Availability

The datasets used and/or analyzed during the current study are available from the corresponding author on reasonable request.
